# Development of a Risk Score to Predict Sudden Infant Death Syndrome

**DOI:** 10.3390/ijerph191610270

**Published:** 2022-08-18

**Authors:** Mounika Polavarapu, Hillary Klonoff-Cohen, Divya Joshi, Praveen Kumar, Ruopeng An, Karin Rosenblatt

**Affiliations:** 1 School of Population Health, The University of Toledo, HH 1010, Mail Stop 119, 2801 W. Bancroft St., Toledo, OH 43606, USA; 2 Department of Kinesiology and Community Health, University of Illinois at Urbana-Champaign, Urbana, IL 61801, USA; 3 Department of Pediatrics, Johns Hopkins All Children’s Hospital, St. Petersburg, FL 33701, USA; 4 Department of Pediatrics, Children’s Hospital of Illinois, Peoria, IL 61603, USA; 5 Brown School, Washington University in St. Louis, St. Louis, MO 63130, USA

**Keywords:** SIDS, prediction model, risk score, risk factors, prevention

## Abstract

Sudden Infant Death Syndrome (SIDS) is the third leading cause of death among infants younger than one year of age. Effective SIDS prediction models have yet to be developed. Hence, we developed a risk score for SIDS, testing contemporary factors including infant exposure to passive smoke, circumcision, and sleep position along with known risk factors based on 291 SIDS and 242 healthy control infants. The data were retrieved from death certificates, parent interviews, and medical records collected between 1989–1992, prior to the Back to Sleep Campaign. Multivariable logistic regression models were performed to develop a risk score model. Our finalized risk score model included: (i) breastfeeding duration (OR = 13.85, *p* < 0.001); (ii) family history of SIDS (OR = 4.31, *p* < 0.001); (iii) low birth weight (OR = 2.74, *p* = 0.003); (iv) exposure to passive smoking (OR = 2.64, *p* < 0.001); (v) maternal anemia during pregnancy (OR = 2.07, *p* = 0.03); and (vi) maternal age <25 years (OR = 1.77, *p* = 0.01). The area under the curve for the overall model was 0.79, and the sensitivity and specificity were 79% and 63%, respectively. Once this risk score is further validated it could ultimately help physicians identify the high risk infants and counsel parents about modifiable risk factors that are most predictive of SIDS.

## 1. Background

Sudden Infant Death Syndrome (SIDS) refers to the death of an apparently healthy infant less than one year of age, whose death remains unexplained after a thorough case investigation, involving the performance of a complete autopsy, examination of the death scene, and review of clinical and medical histories [1,2,3]. Universally, no definition has been accepted, and thus, SIDS is based on a diagnosis of exclusion, where no other known cause of death can be determined [4]. SIDS still remains unacceptably high [5], recorded as the leading cause of post-neonatal mortality, despite the success of the ‘back-to-sleep’ campaign, which reduced SIDS deaths by over 50–80% in the Western world [6,7,8]. During this time period, awareness of SIDS, coupled with sleep position campaigns, breastfeeding recommendations, smoking and environmental tobacco smoke recommendations, changes in death scene investigation protocols [3,9], and improvements in perinatal care also occurred [10], made it difficult to attribute the drop in SIDS solely to the supine sleep practice [9,10].

The peak incidence of SIDS coincides around two to four months of age, and 90% of cases occur before six months of age [11]. Additionally, the prevalence of SIDS is higher in boys than girls, at a 3:2 ratio [12]. In the US, SIDS is much more common in African-American and Native-American infants compared to Caucasian, Hispanic, and Asian infants [6,13,14].

SIDS pathogenesis is a multifactorial condition with a combination of intrinsic and extrinsic factors, such as genetic, environmental, and sociocultural factors [15]. The most widely accepted theory for the causation of SIDS is the ‘triple-risk hypothesis,’ which proposes that SIDS occurs when there is a convergence of three overlapping risk factors—a vulnerable infant, a critical development period, and an exogenous environmental stressor [16]. SIDS occurs in infants with a latent biological vulnerability (i.e., brainstem abnormality or genetic pattern) who are exposed to a trigger event or extrinsic risk factor (e.g., prone sleeping, tobacco smoke) during a critical phase of development [16]. The overlap of these three risk factors is believed to be crucial to causing SIDS [15,17,18].

Both the in utero and the infant’s environment impact the vulnerability to SIDS [19]. This has dramatic effects on two clinical disciplines, obstetrics, and pediatrics [20]. Currently, there are no standardized screening methods to identify infants with intrinsic risk factors, who are at a higher risk of dying from SIDS. Hence, public health efforts have been focused on increasing awareness about extrinsic or environmental risk factors [20]. The ability to accurately identify infants at a higher risk of SIDS facilitates clinical and research efforts for prevention [21].

Currently, there are eight risk score models for SIDS in existence, including the Sheffield birth score (1973) [22], California score (1979) [23], Cardiff score (1982) [24], Oxford score (1985) [25], Sheffield multistage score (1986) [26], Abbreviated Oxford score (1990) [27], New Zealand CID birth score (1990) [27], and the New Zealand CID multistage score (1990) [27], (see Table 1). None of these scores are routinely used in clinical practice, possibly because they are not externally validated and because there are several contemporary known and unknown SIDS risk factors that were never tested in these models.

Hence, the aim of this study is to develop a new SIDS risk scoring system to identify the cumulative effects of maternal, infant, and environmental factors in order to predict the risk of SIDS. This summary measure could ultimately help physicians identify high risk infants and counsel parents about the modifiable risk factors that are most predictive of SIDS reflected in the risk score.

## 2. Methods

### 2.1. Study Population and Data Collection

The original case-control study consisted of a wealth of data with hundreds of variables retrieved from death certificates, parent interviews, and maternal and pediatric medical records [28,29]. The current study utilized unmatched data on 291 SIDS and 242 healthy control infants. The IRB oversight and approval were obtained from the Human Subjects Committee at the University of California, San Diego. The manuscript being submitted only pertains to the secondary data analysis of the de-identified dataset.

During the data collection, the cases were identified based on the death certificates from five health departments located in the Southern California counties of San Bernardino, Riverside, San Diego, Orange, and Los Angeles [28,29]. The SIDS cases had a diagnosis of “classic SIDS”, corresponding to code 798 in the ninth edition of the International Classification of Diseases. They were ascertained by autopsy reports based on the coroners offices’ use of the Autopsy Protocol for Sudden Unexpected Infant Death and Death Scene and the Deputy Coroner Investigation protocols among infants (less than one year of age) who died between 1 January 1989 and 31 December 1992 [28,29]. The control infants were randomly selected from all of the eligible births from the hospitals where the SIDS infants were born [28,29]. The medical records directors contacted the comparison infants’ parents and forwarded the survey packet along with the informed consent. The original data were obtained from a comprehensive telephone interview in English or Spanish, and the information was validated using the mother’s (obstetric) and the infant’s (pediatric) medical records. This information was reported in previous publications [28,29].

### 2.2. Selection of Predictor Variables

The underlying mechanism for our predictor variables is based on the Triple Risk Hypothesis [16]. The selection of our predictor variables was drawn from the literature, eight existing SIDS risk score models, and clinical practice.

The risk factors that appeared in the eight SIDS risk scoring systems, included: maternal age at the time of delivery; infant birth weight; and maternal smoking during pregnancy. Additionally, other potential risk factors from the SIDS literature were evaluated including: gestational age [30]; paternal smoking status during pregnancy [31]; infant’s exposure to passive smoking [32,33]; maternal alcohol use during pregnancy [34]; maternal use of recreational drugs during pregnancy [35]; maternal anemia during pregnancy [36,37]; family history of SIDS [4]; breastfeeding duration [38]; circumcision of the infant [39]; routine sleep position [40,41]; and bed-sharing with parents [42].

The birth weight and gestational age are two important risk factors for SIDS. However, due to the high multicollinearity between these two variables, the accuracy of the statistical models could be potentially compromised if both of the factors were included in the same model [43]. An improvement in the clinical prediction models was suggested by Onland et al., using the birth weight Z scores and gestational age, instead of combining the gestational age and birth weight in the model [43]. Hence, the standardized z-scores were calculated using the STATA software and categorized as “birth weight z score less than −1” and “birth weight z-score more than or equal to −1”. The gestational age in weeks was used as another predictor variable in our statistical analyses.

The maternal age at the time of delivery was measured using two categories: “age below 25 years”, and “age more than or equal to 25 years”. The maternal smoking status was categorized as “mothers who never smoked”, “mothers who were smokers but not during pregnancy”, and “mothers who were smokers and smoked during pregnancy”. Additionally, the variables recorded as “yes” or “no” included: (i) paternal smoking status during pregnancy; (ii) infant’s exposure to passive smoke from mother, father or caretaker; (iii) maternal use of alcohol during pregnancy; (iv) maternal use of recreational drugs during pregnancy; (v) maternal anemia during pregnancy (i.e., hemoglobin levels < 12 g/dL) as determined from medical records; (vi) circumcision of the infant; and (vii) history of SIDS among close and extended family. The breast feeding duration was categorized as “0–2 months”, “2–4 months”, and “more than 4 months”. The infant’s routine sleep position was detailed as “on their stomach”, “on their back”, “on their side”, and “no usual position”. Finally, the infant’s bed-sharing practice with parents was detailed as “no”, “yes”, and “sometimes”.

### 2.3. Statistical Analysis

The descriptive statistics were used to characterize the cases and controls, using mean and standard deviation for the continuous variables and percentages for the categorical variables. All of the analyses were performed using STATA 15.0 statistical software [44].

A multivariable logistic regression model was developed to predict the risk of SIDS among the infants less than one year of age. The initial step of the model development was to examine the relationship between SIDS and each of the risk factors using the study data. Second, the eight statistically significant variables with *p* values less than 0.05 were entered into a stepwise backward selection logistic regression model: the birth weight z scores; maternal age; maternal smoking; paternal smoking during pregnancy; infant’s exposure to passive smoke; anemia during pregnancy; breastfeeding duration; and family history of SIDS. Along with these eight variables, the routine sleep position and bed sharing practices (although not significant) were also entered into the backward stepwise logistic regression model, because of their importance to SIDS. Third, the statistically significant variables with *p* < 0.05 were retained and were entered into the final multivariable logistic regression model. To evaluate the fit of the final logistic regression and its performance, the concordance statistic (C-statistic) was used to assess discrimination, which represented the area under the receiver operating characteristic (ROC) curve. Bootstrap methods were used to obtain a bias-corrected confidence interval for the calculated C-statistic. The calibration was measured statistically using the Hosmer–Lemeshow test.

A post-hoc analysis using two logistic regression models was performed to evaluate the prenatal and postnatal risk factors separately for this multi-factorial disease.

### 2.4. Derivation of SIDS Risk Score

The risk score was developed based on the final logistic regression model, using the regression coefficients as described by Mehta et al. [45]. The appropriate reference category which reflected the minimal risk state for each risk factor was identified and assigned a score of “0” in the scoring system. For example, a breastfeeding duration of greater than four months was chosen as a reference category, signifying it as protective, thereby inferring the least risk, compared to categories of fewer than two months and 2–4 months. Furthermore, these latter categories of individual factors associated with a risk of SIDS (e.g., breastfeeding duration for <2 months and 2–4 months) were assigned scores based on the coefficient from the logistic regression model, rounded to the nearest whole number to produce meaningful risk scores. An individual infant’s total risk score, consisting of both the protective and risk factors, was calculated by adding the individual scores for each risk factor.

## 3. Results

The sample consisted of 533 infants (291 cases and 242 controls), and 38% of the infants were female and 62% were males. All of the racial/ethnic groups for infants were represented, with 42% whites, 12% blacks, 32% Hispanic, 9% Asians, and 4% others. The average birth weight among the control group infants was 3435.56 g (95% confidence interval [CI] = 3371.00–3500.11), and among the SIDS infants was 3221.34 g (95% CI = 3146.42–3296.25). The mean gestational age for the SIDS infants was 39.14 weeks (95% CI = 38.88–39.40), compared to 39.67 weeks for the control group infants (95% CI = 39.48–39.86). The differences in the mean birth weight and gestational age (which are both continuous variables) between the cases and controls using independent sample *t*-tests were statistically significant (*p* < 0.001). A detailed demographic and risk factor distribution among the cases and controls is presented in Table 2 and Table 3.

A total of 13 unadjusted risk factors were examined in relation to SIDS, among which, eight were statistically significant: the birth weight z scores; maternal age; maternal smoking; paternal smoking during pregnancy; infant’s exposure to passive smoke (mother, father, caretaker); anemia during pregnancy; breastfeeding duration; and family history of SIDS (Table 3). The rates of circumcision were comparable between cases (19.8%) and controls (19.6%), and the difference was not statistically significant. The prone sleep position, the most commonly advocated risk factor in the ‘Back to Sleep” campaign, was not statistically significant in our study population. This was because between 1989 and 1992, pediatricians in the US advised infants to be placed in the prone sleep position in order to avoid aspiration [20,46,47]. Thus, approximately 70% of the cases and 61% of the controls were routinely placed on their stomachs during sleep in our study. However, in contrast, the sleep position at the time of death revealed that nearly 60% of the SIDS infants were last placed on their stomachs, and about 69% of the SIDS infants were last found on their stomachs at the time of death. It was impossible to introduce the last sleep position at the time of death into the risk score because there was no comparison information for the healthy controls.

### 3.1. Our Risk Score Model

**Figure 1:**
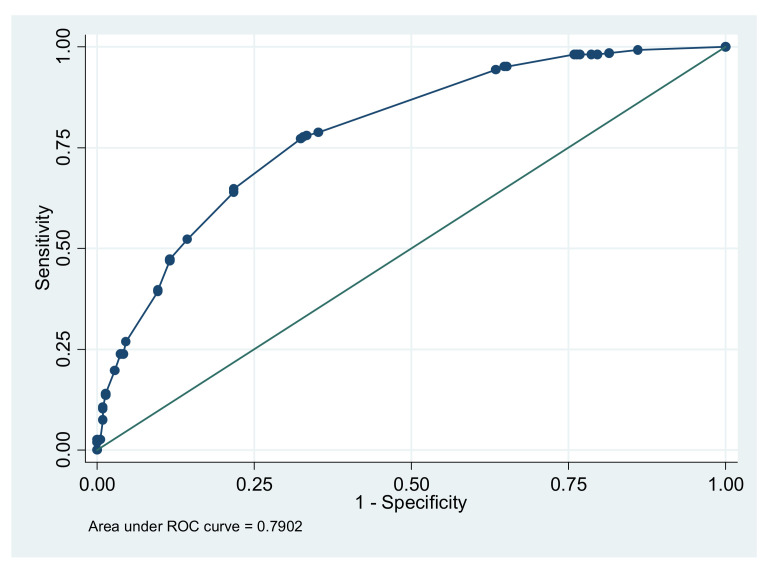
Area under the receiver operating characteristic curve (AUC) for the risk score model.

The final logistic regression model to predict the risk of SIDS is presented in Table 4. There were six significant risk factors in the final model. The factor that was associated with the highest risk of SIDS death was breastfeeding for less than two months (odds ratio [OR] = 13.85, 95% CI = 5.25–36.55, *p* < 0.001) compared to those who were breastfed for over four months. The other five statistically significant variables, in descending order of strength of their odds ratios, were: family history of SIDS (OR = 4.31, 95% CI = 2.24–8.31); infant birth weight of ≤1 SD below the mean (OR = 2.74, 95% CI = 1.41–5.33); exposure of the baby to passive smoking (OR = 2.64, 95% CI = 1.65–4.20); maternal anemia during pregnancy (OR = 2.07, 95% CI = 1.09–3.94); and maternal age of less than 25 years at the time of childbirth (OR = 1.77, 95% CI = 1.13–2.79). The model fit the data reasonably well in terms of discrimination (C-statistic = 0.79 [95% CI = 0.76–0.84]) (Figure 1) and calibration (Hosmer–Lemeshow goodness-of-fit test, *p* = 0.83).

Based on the final multivariable logistic model, a risk score system was developed to predict an infant’s risk for SIDS. In reference to Table 3, for each variable, the category with the lowest risk for SIDS was assigned a risk score of “0”. The remaining category’s coefficient was rounded to the nearest whole number in order to assign a risk score. The risk score for each variable ranged from 0 to 3 (Table 4), and each infant’s total risk score ranged from 0 to 9.

### 3.2. Performance of Our Risk Score Compared to Existing Risk Scores for SIDS

The sensitivity of the overall logistic regression model calculated using the predicted risk of SIDS was 79.2%, and the specificity was 63.0%. The area under the curve for this model was 0.79 (Figure 1). The areas under the curve were not available for the other existing eight risk scores.

A total of 13 variables (e.g., infant’s exposure to passive smoking, duration of breastfeeding, maternal anemia during pregnancy, circumcision) which are contemporary, were tested in contrast to the existing models. The threshold for the total cut-off risk score of six and above (i.e., scores 6, 7, 8, and 9) was best at predicting SIDS, with a sensitivity of 44% and specificity of 90%, based on 291 SIDS cases.

The specificity in our model was the highest of the eight existing risk score models (Table 5). Among all of the risk score models, the Oxford score reported the highest sensitivity at 70%; albeit for 34 SIDS cases. Additionally, the sensitivity for the Sheffield score was 62% for 34 SIDS cases and the sensitivity was 53% among 34 SIDS cases for the California score. A detailed comparison of our risk scoring system with the existing models is presented in Table 5.

### 3.3. Post-Hoc Analysis

A logistic regression model for **prenatal risk factors** consisted of: (i) maternal smoking during pregnancy (never smoked [reference category], smoker but did not smoke during pregnancy, smoker and smoked during pregnancy); (ii) family history of SIDS; (iii) maternal age during pregnancy (<25 years, ≥25 years); (iv) anemia during pregnancy; (v) alcohol consumption during pregnancy; and (vi) recreational drugs during pregnancy. The statistically significant risk factors were: (i) family history of SIDS (OR = 4.19, 95% CI: 2.31–7.59); (ii) anemia during pregnancy (OR = 2.29, 95% CI: 1.29–4.08); and (iii) maternal smoking (never smoked [reference category], smoker but did not smoke during pregnancy [OR = 1.69, 95% CI: 1.01–2.84], smoker and smoked during pregnancy [OR = 2.01, 95% CI: 1.23–3.31]).

A separate logistic regression model for the **postnatal risk factors** included: (i) infant exposure to passive smoking (mother, father, or caregiver); (ii) birthweight z score; (iii) breastfeeding duration (<2 months, 2–4 months, >4 months); (iv) circumcision; (v) routine sleep position; and (vi) bedsharing. The risk factor with the highest odds ratio was breastfeeding duration (>4 months (reference category), 2–4 months OR = 5.12, CI: 1.72–15.23, <2 months OR = 16.42 CI: 6.30–42.80). When the breastfeeding duration was collapsed into two categories, the odds ratio for infants’ breastfeeding for <2months was 6.21 (95 CI: 3.71–10.41), when compared to the infants who breastfed for more than 2 months. The infants exposed to passive smoking were 2.92 times (95% CI: 1.88–4.55) more likely to die of SIDS compared to those who were not exposed.

## 4. Discussion

In this study, a risk scoring system was developed to predict the risk of SIDS among infants less than one year of age using data from a case-control study consisting of 294 cases and 242 controls. This is the first statistical risk-scoring model that was developed from a rich set of maternal, paternal, and infant characteristics along with environmental factors. In contrast to the existing eight risk score models, our model was the first to test passive tobacco smoke, sleep position, and circumcision.

Our study sample resembled a typical SIDS population in California consisting of a high percentage of males (62%) who were racially/ethnically diverse. Additionally, the majority of infants died before 6 months of age (89%). All of the patient responses were validated with medical records (of mothers and babies), and infant death certificates, establishing the internal validity and reliability of the data.

The variables in our final risk score were low birth weight, family history of SIDS, young maternal age, infants’ exposure to passive smoking, maternal anemia, and breastfeeding duration. Only one previous risk-scoring model, Oxford, evaluated family history, notating it as “previous SIDS” [25]. In our model, family history was strongly associated with SIDS (OR = 4.31). Furthermore, the breastfeeding duration was not included in any previous risk-scoring models. Rather, breastfeeding intention was evaluated in Sheffield’s birth [22] and multi-stage [26] scores, while infant feeding was assessed in Cardiff’s scores [24], and feeding change within the first month of life was measured in the New Zealand CID multi-stage score [27].

In our study, breastfeeding duration for less than two months or 2–4 months, resulted in corresponding odds ratios of 6.11 and 13.85, respectively, when compared to greater than 4 months. Our classification of breast feeding duration was based on a previous meta-analysis that assessed the associations between breastfeeding duration and SIDS, using data from eight case control studies [38]. They reported that a breastfeeding duration of at least 2 months was associated with half of the risk of SIDS [38]. In our study, the magnitude of the protective effect of breastfeeding increased with a longer duration. A plausible mechanism for the protective effect is that breastfed infants are more easily aroused from sleep than formula-fed infants, a phenomenon which is critical in infants with intrinsic vulnerability to SIDS [38,51,52,53]. The WHO recommends exclusive breastfeeding until 6 months; however, this target will require significant compliance by new mothers in developed countries [54]. In fact, any breastfeeding (i.e., breastfeeding in combination with supplemental feeding) is also beneficial for protection against SIDS [54], particularly for mothers who produce insufficient amounts of milk and require alternatives in conjunction with breast milk. Fortunately, the reduction in SIDS risk appears to occur after only two months of any type of breastfeeding (e.g., exclusive, supplemental) [54]. Another study by Klonoff-Cohen et al. showed that breast-feeding was protective for SIDS among nonsmokers (OR = 0.37) but not smokers (OR = 1.38), when adjusted for potential confounders [55].

Moving forward, breastfeeding “intention” rather than breastfeeding “duration” should be used to predict SIDS prospectively. This is because actual breastfeeding duration (i.e., 2 months, 4 months) may not be possible to achieve for babies if they expire from SIDS before the two time points.

To date, this is the first risk model to evaluate maternal anemia as a risk factor for SIDS. The maternal anemia may lead to SIDS via a fetal hypoxia mechanism [55], resulting in a disturbance in the fetal nervous system or lung development. The odds ratio for maternal anemia in our model was 2.07.

Finally, passive smoking has never been evaluated in the previous risk models, only maternal smoking, which was included in the California [23] and Cardiff [24] risk models. In our model, the infant’s exposure to passive smoking was defined as the mother, father, or caretaker smoking in the same room as the infant, resulting in a corresponding odds ratio of 2.64.

A meta-analysis of 35 case-control studies revealed that maternal smoking during pregnancy was a strong risk factor for SIDS (OR = 2.25 [95% CI 2.03–2.50]) [56]. However, in our final risk score model, it was not significant. A possible explanation for this occurrence is that we categorized maternal smoking differently (i.e., into mothers who never smoked, mothers who were smokers but not during pregnancy, and mothers who were smokers and smoked during pregnancy) compared to other researchers, who stratified according to no cigarette use and cigarette use during pregnancy [57]; or never smoked or quit early in the pregnancy vs. continued or quit late [58]. When both the pre- and post-natal factors were combined in our risk model, passive smoking was a stronger predictor than maternal smoking during pregnancy, ultimately resulting in its elimination in backward stepwise logistic regression. Nevertheless, smoking during pregnancy should be strongly advocated as a risk factor when physicians are advising couples about the risks of SIDS. As prenatal and postnatal smoking are now two of the largest potentially modifiable risk factors for SIDS, pregnant women should be motivated and supported to stop smoking, and protect themselves from exposure to second- and third-hand tobacco [59].

The low birth weight infants are more prone to SIDS because of the prematurity of the regulatory pathways of breathing and circulation [60]. The higher risk of infants born to mothers of an age less than 25 years has been attributed to several factors, including different attitudes to child care practices compared to older mothers [61]. Additionally, teenage pregnancies are at high risk for low birth weight infants and are less likely to breastfeed [61,62,63].

Three statistically nonsignificant variables require a comment. Circumcision (i.e., non-emergency), a novel risk factor for SIDS which was explained by a wear-and-tear hypothesis, was initially proposed by Elhaik in 2016 [39]. A statistically significant association between SIDS trends and male neonatal circumcision rates was subsequently revealed in an ecologic study of fifteen countries between 2004–2013 [64]. In contrast, our study found that the risk associated with circumcision was not significant among male SIDS infants compared to those in the control group (i.e., 34% vs. 31%, *p* = 0.64). During our data collection process, information on neonatal circumcision was obtained from medical records, thereby increasing the internal validity of our findings. However, due to a relatively small subsample of male circumcised infants (i.e., 19.8% of cases and 19.6% of controls), as well as missing circumcision data, we did not find an effect. Further large epidemiological studies with data on individual infants are required to confirm or refute circumcision as a risk factor for SIDS.

The routine sleep position was not significant in our model. This dataset was collected before the Back to Sleep Campaign. At that time, pediatricians in the US recommended that infants be placed in prone sleep position in order to decrease the likelihood of aspiration, gastroesophageal reflux, as well as to improve pulmonary function and sleeping [20,46,47]. Hence, in this dataset, there was almost an identical prevalence (i.e., 66% vs. 64% for cases and controls, respectively) of case and control infants routinely placed on their stomachs. Thus, in a previous analysis on the same dataset, there was no difference in the routine sleep position for SIDS infants and control infants (OR = 0.76, 95% CI = 0.42–1.38), while simultaneously adjusting for birth weight, medical conditions at birth, breastfeeding, passive smoking, maternal recreational drug use, prenatal care, and infant vomiting [55]. However, death scene sleep positions (e.g., last placed and last found) were statistically significant for prone sleep position, where 80% of cases were found sleeping on their abdomens at the time of death [55]. The supine sleeping position should always be discussed by pediatricians when advising couples about the risks of SIDS in their newborn infants.

The sleep environment is also important in this multifactorial disease. Due to the efforts to improve the sleep environment and reduce exposure to tobacco smoke, the rate of SIDS has fallen significantly. Nevertheless, SIDS still remains a major contributor to post-neonatal mortality in the United States, accounting for approximately 1389 annual deaths [6].

Bedsharing was also not significant in our risk score model. In a previous study, significant differences between bed sharing among African-American and Latin-American parents compared with white parents were reported, with no significant relationship between routine bedsharing during the daytime or night-time and SIDS [55]. Similarly, a study conducted with 400 SIDS infants and 1386 controls from five English health regions reported that bed-sharing was not significantly associated with SIDS when the infant was not co-sleeping on a sofa, not sleeping next to a parent who drank more than two units of alcohol or was a smoker [65]. An analysis of the risk factors by Trachtenberg et al. in the pre- (1991–1993) and post-“Back to Sleep” (1996–1998) campaign revealed that last-placed and last-found sleep positions and bedsharing significantly differed between the case and control infants during these two time periods [66].

### Strengths and Limitations

One of the major limitations of our study is that the SIDS cases and controls were collected from death certificates, parent interviews, and maternal and pediatric records from 1989–1992, prior to the “Back to Sleep” campaign. Thus, in this study, we conducted a retrospective analysis which may be prone to recall or misclassification bias or result in an inferior level of data compared to a prospective study design. Nevertheless, the criteria used to diagnose SIDS cases were stringent. The definition of SIDS in our study is in contrast to the current CDC and AAP definition of sudden unexpected infant death, which combines SIDS with undetermined, asphyxia, ill-defined, or unknown causes, and accidental suffocation and strangulation. Developing effective preventive measures for SIDS requires a diagnostic method that enables the distinction of SIDS from SUID [67]. We had the advantage of focusing only on SIDS in our model, which needs to be confirmed in cases diagnosed within the past decade in order to confirm the relevance to current clinical practice.

In the literature, several genetic mutations or polymorphisms associated with SIDS were described, including cardiac defects, abnormalities in the serotonin pathway, dysregulation of the inflammatory response, inborn errors of metabolism, and changes in nicotine receptors [15]. None of these genetic mutations were collected in our study nor tested in the eight risk score models.

The circumcision data were ascertained in-hospital. Hence, those circumcisions performed later in life (e.g., 8th day) were unknown, and this would be another limitation of our study. The data were missing on the circumcision variable in 41.8% of the male SIDS infants and 36.5% of the male control infants, resulting in non-differential misclassification and biasing the data towards the null.

A post-hoc analysis was performed to independently evaluate the pre-and post-natal risk factors. It was important to delineate these risks separately because SIDS encompasses both obstetricians’ and pediatricians’ expertise when caring for the mother, fetus, and infant, prior to a SIDS event. The most important prenatal risk factors were: (i) family history of SIDS; (ii) anemia during pregnancy; and (iii) maternal smoking. For the postnatal risk factors, breastfeeding duration, infant exposure to passive smoking, and birth weight had the highest odds ratios.

In conclusion, the specificity in our model was the highest of all of the existing risk scores using cut-off scores. While the major research priority has been to obtain high sensitivity and specificity for any test, optimizing both with a single test has always been a challenge [68]. The population screening for rare diseases requires high specificity in order to reduce the number of false-positive results to an acceptable level [68,69]. Our sensitivity for 291 SIDS cases was 44% with a threshold cutoff total score of ≥6. Higher sensitivities were previously reported by the Oxford score (70%), Sheffield score (62%), and California score (53%) (Table 5). All three of these risk scores utilized the same set of 34 SIDS cases.

## 5. Conclusions

Although a good risk score can predict adverse outcomes of SIDS on average, it is not designed to precisely predict a single patient’s risk. The reason that previous scoring schemes may not be embraced is likely due to the reality that busy clinicians may not have time to calculate them in a well-child visit. Nevertheless, our risk scoring system must be further validated with a future national or international dataset that contains a multitude of risk factors that are similar to our study. Our risk score could be used as a tool to help physicians and their patients make informed decisions for SIDS prevention, explicitly targeting infants at high risk, such as families who had encountered previous SIDS deaths, who practiced no breastfeeding, and who exposed their infants to passive smoke.

## Figures and Tables

**Table 1:** SIDS Risk Scores from the Literature.

Risk Score	Variables
Sheffield birth score [22]	Mother’s age
	Previous pregnancies
	Twin pregnancy
	Mother’s blood group
	Urinary infection during pregnancy
	Duration of 2nd stage labor
	Birthweight
	Feeding intention at the time of delivery
California score [23]	Maternal age < 25 years
	Maternal smoking
	Pregnancy < 12 months
	<11 perinatal visits
	Birthweight < 3000 g
	Gestational age < 4 0 wk
	Male sex
	Blue collar family
Cardiff score [24]	Age of mother at birth
	Maternal smoking
	Antenatal clinic visits
	Gestation at booking
	Previous deliveries
	Pregnancy complications
	2nd stage of labor
	Admission to SCBU
	Mode of delivery
	Birthweight
	Maternal birth injury
	Season of birth
	Sex of infant
	Infant feeding
	Area of residence
	Social class
	Mother’s employment
	Employment of partner
Sheffield multistage score [26]	Mother’s age
	Birth order
	Twin
	Blood group A
	Urinary infection during pregnancy
	Duration 2nd stage labor
	Birthweight
	Breastfeeding intention at delivery
Oxford score [25]	Mother’s age at delivery and birth order
	Maternal drug addiction
	Mother took barbiturates
	Previous SIDS
	Interpregnancy interval
	Infection during pregnancy
	Multiple births/twins
	Gestation
	Marital status
	Husband’s social class
Abbreviated Oxford score [27]	Maternal age and parity
	Infants’ birthweight
	Marital status
New Zealand CID birth score [27]	Maternal age
	Parity
	Infants’ birthweight
	Marital status
New Zealand CID multistage score [27]	Birth score
	Mother’s age
	Parity
	Birth weight
	Marital status
	1-month score
	Birth score
	Feeding change
	Comments on home

**Table 2:** Demographics.

Characteristics	SIDS No. (%)	Controls No. (%)
Gender		
Females	109 (37.46)	94 (38.84)
Males	182 (62.54)	148 (61.16)
Race/Ethnicity		
White	117 (40.21)	107 (44.21)
Black	38 (13.06)	27 (11.16)
Hispanic	92 (31.62)	80 (33.06)
Asian	27 (9.28)	22 (9.09)
Others	17 (5.84)	6 (2.48)

**Table 3:** Risk Factors Associated with SIDS.

Risk Factor	SIDS No. (%)	Controls No. (%)	*p*
**Birth weight z-score**			
More than −1	55 (19.3)	16 (6.93)	Reference
Less than or equal to −1	230 (80.7)	215 (93.07)	<0.001 *
**Maternal age**			
More than or equal to 25 years	156 (54.17)	173 (73.31)	Reference
Less than 25 years	132 (45.83)	63 (26.69)	<0.001 *
**Maternal smoking**			
Never smoked	140 (48.11)	156 (64.46)	Reference
Smoker but not during pregnancy	56 (19.24)	39 (16.12)	0.049
Smoker and smoked during pregnancy	95 (32.65)	47 (19.42)	<0.001 *
**Paternal smoking during pregnancy**			
No	180 (61.86)	196 (80.99)	Reference
Yes	110 (37.8)	46 (19.01)	<0.001 *
**Infants’ exposure to passive smoking**			
No	166 (57.24)	196 (81.67)	Reference
Yes	124 (42.76)	44 (18.33)	<0.001 *
**Maternal use of recreational drugs during pregnancy**			
No	268 (92.17)	233 (96.28)	Reference
Yes	22 (7.59)	9 (3.72)	0.06
**Maternal use of alcohol during pregnancy**			
No	197 (67.93)	153 (63.93)	Reference
Yes	93 (32.07)	89 (36.78)	0.26
**Anemia during pregnancy**			
No	239 (82.13)	216 (89.26)	Reference
Yes	52 (17.87)	26 (10.74)	0.02 *
**Breastfeeding duration**			
More than 4 months	5 (1.77)	58 (23.97)	Reference
2–4 months	19 (6.74)	39 (16.12)	<0.001 *
Less than 2 months	258 (91.49)	145 (59.92)	0.001 *
**Family history of SIDS**			
No	210 (74.47)	216 (92.7)	Reference
Yes	72 (25.53)	17 (7.3)	<0.001 *
**Co-sleeping**			
No	188 (65.51)	173 (71.49)	Reference
Yes	65 (22.65)	49 (20.25)	0.36
Sometimes	34 (11.85)	20 (8.26)	0.14
**Circumcision in male infants (*n* = 330)**			
No	70 (38.46)	65 (43.92)	Reference
Yes	36 (19.78)	29 (19.59)	0.64
Missing data	76 (41.76)	54 (36.49)	
**Routine sleep position**			
Stomach	182 (62.98)	147 (61)	Reference
Side	24 (8.3)	19 (7.88)	0.95
Back	37 (12.8)	30 (12.45)	0.99
No usual position	44 (15.22)	45 (18.67)	0.32

* Statistically significant at the alpha level of 0.05. All risk factors were categorical and used separate logistic regression models.

**Table 4:** Final Prediction Model for SIDS and Risk Scores.

Variable	Coefficient	Odds Ratio (95% Confidence Interval)	*p* Value	Risk Score
**Birth weight z score**				
More than or equal to −1		Reference		0
Less than −1	1.0	2.74 (1.41 to 5.33)	0.003 *	1
**Passive smoke**				
No exposure		Reference		0
Exposed	1.0	2.64 (1.65 to 4.20)	<0.001 *	1
**Maternal age at the time of childbirth**				
More than or equal to 25 years		Reference		0
Below 25 years	1.0	1.77 (1.13 to 2.79)	0.01 *	1
**Family History of SIDS**				
No				0
Yes	1.5	4.31 (2.24 to 8.31)	<0.001 *	2
**Breastfeeding duration**				
More than 4 months		Reference		0
2–4 months	1.8	6.11 (1.99 to 18.73)	0.002 *	2
Less than 2 months	2.6	13.85 (5.25 to 36.55)	<0.001 *	3
**Anemia during pregnancy**				
No		Reference		0
Yes	1.0	2.07 (1.09 to 3.94)	0.03 *	1

* Statistically significant at the alpha level of 0.05.

**Table 5:** Performance of Our Risk Score Compared to Existing Risk Scores.

Risk Score	Author	Population	Cutoff Score	Sensitivity	Specificity
Our model		291 SIDS infants and 242 healthy controls from Southern California, 1989–1992	6	44%	90%
Sheffield birth score [22]	Peters [48]	34 SIDS and 318 controls from British Birth Cohort, 1970	492	62%	80%
	Cameron [26]	61 SIDS and 131 controls from Melbourne, 1980	500	43%	89%
	O’Brien [49]	48 SIDS and 192 controls from Dublin, 1979–1981	500	29%	85%
	Brooks [50]	123 SIDS and 637 controls from England, 1983–1987	500	35%	89%
California score [23]	Peters [48]	34 SIDS and 318 controls from British Birth Cohort, 1970	5	53%	80%
Cardiff score [24]	Murphy [24]	99 SIDS cases from the 47,413 live births from the Cardiff Births Survey	102	NA *	NA *
Sheffield multistage score [26]	Cameron [26]	61 SIDS and 131 controls from Melbourne, 1980	754	44%	85%
	O’Brien [49]	48 SIDS and 192 controls from Dublin, 1979–1981	745	38%	85%
Oxford score [25]	Peters [48]	34 SIDS and 318 controls from British Birth Cohort, 1970	1.745	70%	80%
	Brooks [50]	123 SIDS and 637 controls from England, 1983–1987	1.745	57%	77%
Abbreviated Oxford score [27]	Nelson [27]	377 possible preventable post neonatal deaths and 936 controls from New Zealand, 1979–1984	1.44	50%	77%
New Zealand CID birth score [27]	Nelson [27]	377 possible preventable post neonatal deaths and 936 controls from New Zealand, 1979–1984	342	50%	79%
New Zealand CID multistage score [27]	Nelson [27]	514 consecutive births in 1986 and 49 SIDS from New Zealand, 1986–1987	355	50%	80%

* NA: Not available.

## Data Availability

The data presented in this study are available on request from the corresponding author. The data are not publicly available due to the sensitivity of the information.
